# Urinary Strong Ion Difference as a Marker of Renal Dysfunction. A Retrospective Analysis

**DOI:** 10.1371/journal.pone.0156941

**Published:** 2016-06-03

**Authors:** Paolo Balsorano, Stefano Romagnoli, Samuel Kagan Evans, Zaccaria Ricci, Angelo Raffaele De Gaudio

**Affiliations:** 1 Department of Health Sciences, Section of Anaesthesiology, Intensive Care and Pain medicine, University of Florence, Florence, Italy; 2 Department of Pulmonary and Critical Care Medicine, Rhode Island Hospital, Providence, United States of America; 3 Department of Cardiology and Cardiac Surgery, Pediatric Cardiac Intensive Care Unit, Bambino Gesù Children's Hospital, IRCCS, Rome, Italy; University of Sao Paulo Medical School, BRAZIL

## Abstract

**Introduction:**

The kidneys play a crucial role in the regulation of electrolytes and acid-base homeostasis. Urinary Strong Ion Difference (SIDu = NaU + KU—ClU) represents an important aspect of renal acid-base regulation. We evaluated the role of SIDu as a marker of renal dysfunction in critically ill patients.

**Materials and Methods:**

Patients admitted to the Medical Intensive Care Unit with a diagnosis of AKI for whom concomitant urinary samples available for SIDu calculation were retrospectively reviewed and staged according to KDIGO criteria for 3 days from inclusion. Patients were classified as Recovered (R-AKI) or Persistent-AKI (P-AKI) whether they exited KDIGO criteria within the 3-day observation period or not. A control group with normal renal function and normal serum acid-base and electrolytes was prospectively recruited in order to identify reference SIDu values.

**Results:**

One-hundred-and-forty-three patients with a diagnosis of AKI were included: 77 with R-AKI, and 66 with P-AKI. Thirty-six controls were recruited. Patients with P-AKI had more severe renal dysfunction and higher mortality than patients with R-AKI (SCr 2.23(IQR:1.68–3.45) and 1.81(IQR1.5–2.5) mg/dl respectively, p<0.001; 24-h UO 1297(950) and 2100(1094) ml respectively, p = 0.003); 30-d mortality, 39% and 13% respectively; p<0.001). SIDu significantly differed between groups, with rising values from controls to P-AKI groups (16.4(12), 30(24) and 47.3(21.5) mEq/l respectively, p<0.001).

**Discussion:**

SIDu may be a simple and inexpensive tool in AKI patients’ evaluation. Further research is needed to evaluate the ability of SIDu to identify patients with renal dysfunction before derangements in serum creatinine or urine output are observed.

## Introduction

Despite increased awareness by clinicians, Acute Kidney Injury (AKI) remains a serious clinical condition with high morbidity and mortality[1,2]. Current diagnostic criteria are based on serum Creatinine (SCr) and urinary output (UO)[3,4,5,6]. However, a growing body of evidence suggests that these markers may be insufficient in the timely identification of kidney injury and may lead to delays in diagnosis and treatment[7,8,9].

The evidence for novel serum and urinary biomarkers of AKI in critically ill patients is lacking [10,11,12,13,14]. Kidneys play a crucial role in the regulation of electrolytes and acid-base homeostasis[15,16]. Urinary Anion Gap ([AGu = [Na+]u + [K+]u–[Cl–]u) is traditionally used in the diagnosis of hyperchloremic metabolic acidosis, namely in differentiating renal from non renal causes. In the quantitative physicochemical approach to acid-base disorders originally described by Stewart, AGu is replaced by the Urinary Strong Ion Difference (SIDu) [17,18,19]. AGu and SIDu are mathematically equivalent. Hence, kidney dysfunction could be manifested by the early inability to regulate acid-base disturbances caused by critical illness. In patients with metabolic acidosis, impaired renal function was associated with greater SIDu[20,21,22]. Furthermore, higher SIDu values were found in patients who developed AKI[23].

The aim of the present study was to investigate SIDu as a marker of renal dysfunction in critically ill patients with AKI.

## Materials and Methods

A retrospective study was conducted in the Medical Intensive Care Unit (MICU) of the Rhode Island Hospital (Providence,RI, US). All patients admitted between September 2012 and September 2013 with a diagnosis of AKI according to KDIGO criteria, at any moment of their ICU stay, and concomitant urinary samples available for SIDu calculation were reviewed. The Rhode Island Hospital Institutional Review Board approved the study and waived the need for informed written consent due to its retrospective nature. Patients’ records were anonymized and de-identified prior to analysis.

For the purpose of the study we excluded patients with unbalanced acid-base and/or electrolytes homeostasis, hematuria, renal transplant, need for bladder irrigation, prior creation of a neo-bladder, pregnancy, end stage renal disease, and age less than18 years. Patients were included if serum and urine chemistries had been withdrawn within one hour of each other.

Patients demographics, diagnosis, severity scores (SAPS 2), 24-h UO, use of vasopressors, loop diuretics, need for mechanical ventilation and renal replacement therapy (RRT) during the observation period as well as ICU length-of-stay and 30-day mortality were recorded.

In order to identify normal SIDu values, a control group with normal renal function was subsequently and prospectively recruited. All patients admitted between May and June 2015 with normal renal function defined by the lack of any criteria compatible with any KDIGO stage were included. We excluded patients with hematuria, bladder irrigation, neo-bladder, pregnancy, end stage renal disease, and age less than 18 years.

### AKI definition

Patients were staged according to KDIGO criteria. Baseline SCr was defined as the lowest SCr in the previous 6 months, or if not available, the plasma creatinine nadir during the ICU stay. Patients were staged according to both SCr and UO criteria (Table 1).

**Table 1:** KDIGO criteria for AKI diagnosis.

Stage	Serum creatinine	Urine Output
1	1.5–1.9 times baseline	<0.5 ml/kg/h for 6–12 hours
	OR	
	≥ 0.3 mg/dl(≥26.5 μmol/L) increase	
2	2.0–2.9 times baseline	<0.5 ml/kg/h for ≥12 hours
3	3.0 times baseline	< 0.3 ml/kg/h for ≥24 hours
	OR	OR
	Increase in serum creatinine to ≥4.0 mg/dl(≥353.6 μmol/L)	Anuria for ≥12 hours
	OR	
	Initiation of RRT	
	(in patients <18 y, decrease in eGFR to <35ml/min/1.73m2)	

Abbreviations: eGFR, estimated GFR; RRT, renal replacement therapy.

Day 0 was defined as the day when AKI was present and urinary biochemistry was available, which did not necessarily correspond to AKI onset day. All AKI patients were followed and staged according to KDIGO criteria for the next 3 days following inclusion. Controls were followed for 1 day. Patients who no longer met KDIGO criteria within the 3-day observation period were classified as Recovered-AKI (R-AKI); patients who still met KDIGO criteria within this timeframe were classified as Persistent-AKI (P-AKI).

### Laboratory measures

Recorded measures included arterial blood gases, serum lactate, serum urea, creatinine (SCr), Na^+^, K^+^, Ca^2+^, Mg^2+^, Cl^-^, phosphate and albumin if available. Day-0 urinary Na^+^ (NaU), K^+^ (KU), Cl^-^ (ClU) were recorded (urine analysis were performed on samples of the collecting bags which were routinely emptied every 4 hours). Derived variables included:

- *SIDu* = *NaU* + *KU* − *ClU*;
- *Plasmatic apparent SID* (*SIDa*) = [*Na*+] + [*K*+] + [*Ca*2+] + [*Mg*2+] − [*Cl*−];
- *Plasmatic effective SID* (*SIDe*) = [*HCO*3−] + [*albumin*−] + [*Pi*−].
  [Albumin-] and [Pi-] (mmol/L) were calculated from measured values and pH by the following equations:
  [albumin−]=[albumin](0.123pH–0.631)
  [Pi−]=[Pi](0.309pH–0.469).

### Statistical analysis

Data were analyzed with MedCalc (v12.2.1). Metric data were tested for normal distribution with the Kolmogorov-Smirnov test. Results are expressed as mean and standard deviations (SD), or median and interquartile range (IR) as appropriate. Data were compared using the t-test or the Mann-Whitney U test where appropriate. Categorical variables were compared using Chi-square or Fisher exact test. ANOVA or Kruskal Wallis test were used to compare multiple means or medians respectively.

Receiver Operating Characteristic Curve (ROC) analysis was performed in order to assess day-0 urinary Strong Ion Difference(SIDu) diagnostic performance to discriminate controls (No-AKI) and P-AKI patients, and R-AKI and P-AKI patients respectively.

Patients enrollment in our retrospective study depended upon availability of simultaneous serum and urine samples in AKI patients. We calculated, however, that this study had a 95% power to detect a difference between means of 13.81 with a significance level (alpha) of 0.05 (two-tailed).

## Results

One-hundred-and-forty-three patients with a diagnosis of AKI were included: 95 were males (66%) and 48 females (34%). Mean age was 63 (16.3) yrs. Thirty-six control patients were recruited. Patients' characteristics are summarized in Table 2.

**Table 2:** Baseline characteristics between patients with Recovered (R-), Persistent (P-) AKI and Control Group.

	R-AKI	P-AKI	Control	p
Patient characteristics				
Gender(M/F)	53/24	42/24	22/14	0.6
Age yrs(SD)	62.3(16.7)	63(15.9)	66.4(12.50	0.59
Baseline creatinine(mg/dl)(IQR)	0.92(0.75–1.12)	1.07(0.84–1.3)	0.78(0.5–0,8)	<0.05*
SAPS 2 (SD)	40.4(12)	52.5(17)	29.5(14)	<0.05*
Reason for ICU admission(%)				
Acute respiratory failure	13(16)	8(12)	8(22)	0.65
Septic shock	21(27)	23(34)	3(8)	0.46
Cardiovascular failure	4(5)	2(3)	0(0	0.85
Liver Failure	3(4)	4(6)	0()	0.87
Hemorragic shock	8(10)	5(7)	0()	0.73
Neuro	1(1)	6(9)	4(11)	0.1
Other	27(35)	18(27)	21(58)	0.2
Treatments(%)				
Vasoactive drugs	11(16)	23(34)	3(8)	<0.05*
Renal Replacement Therapy	0(0)	8(12)	0()	<0.05*
Diuretics	10(13)	26(39)	3(8)	<0.05*
30-d mortality(%)	10(13)	26(39)	3(8)	<0.05*
LOS-ICU days(IQR)	4(2–8)	4.5(2–13)	3(1–6)	0.51

Results reported as means(SD), medians(interquartile range) or n(%). SAPS 2, Simplified Acute Physiology Score 2. p values are for comparison across the three groups.

* is for variables that differ statistically across groups of patients.

### Recovered vs persistent AKI

Seventy-seven (53%) patients were classified as R-AKI, while 66 (47%) patients as P-AKI. Baseline SCr and SAPS2 score were higher in P-AKI than R-AKI group (p<0.05 and <0.05 respectively). Patients with P-AKI required more diuretics, vasopressors and need for RRT than R-AKI patients (p<0.05, 95% CI: 10.8–40.4; p<0.05, 95% CI: 5.1–34.4; p< 0.05, 95% CI: 3.8–22.3 respectively). Mortality was higher in P-AKI group than R-AKI group (13% vs 39% respectively; p<0.05; 95% CI: 11.9–41.3).

### Renal function and SIDu

**Fig 1:**
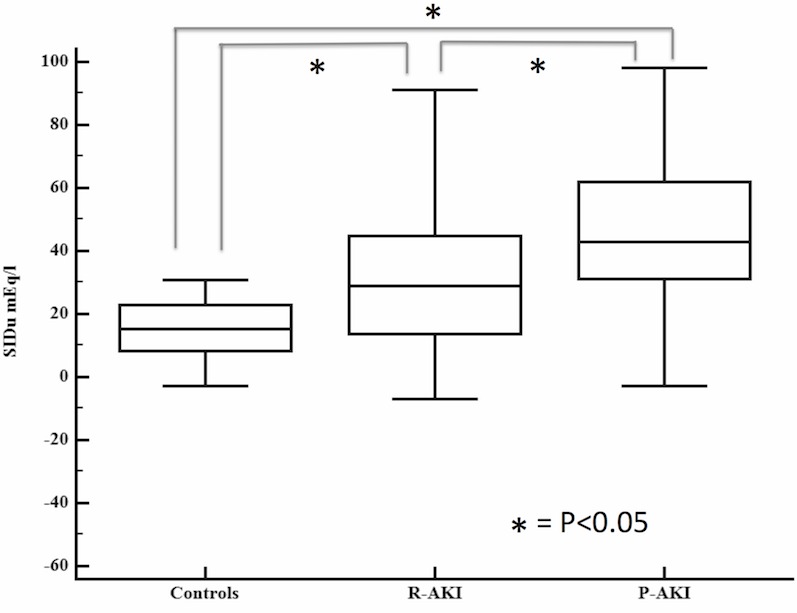
Boxplot representation of day-0 urinary Strong Ion Difference values across the three groups.

Patients with P-AKI had more severe renal dysfunction than patients with R-AKI: SCr values were 1.81 (IQR:1.5–2.5) and 2.23 mg/dl(IQR:1.68–3.45) (p<0.001), while UO was 1297(SD:950) ml and 2100(SD:1094) respectively (p = 0.003). R-AKI group showed less severe renal dysfunction than P-AKI group according to KDIGO classification: KDIGO-1 and 2–3 were diagnosed in 41 and 36 R-AKI patients respectively, whereas 20 KDIGO1 and 47 KDIGO2-3 patients were observed in the P-AKI group (OR 2.7, 95% CI 1.3–5.3, p = 0.004). SIDu values significantly differed between R-AKI and P-AKI groups (p<0.0001). Plasmatic SIDa did not differ between control, R-AKI and P-AKI patients (38.3(SD:3.2), 38.8(SD:6.8), and 38.2(SD:4.9) mEq/l respectively; p = 0.9). On the contrary, SIDu significantly differed between groups (16.4(SD:12), 30(SD:24) and 47.3(SD:21.5) mEq/l respectively), with rising values from No-AKI to P-AKI groups (p<0.001) (Table 3) (Fig 1).

**Table 3:** Day-0 renal function and Strong Ion Difference values of patients without acute kidney injury(Control Group), with reversible AKI(R-AKI) and persistent AKI(P-AKI).

	R-AKI	P-AKI	Controls	p
Renal function				
Urinary Output(ml/24h)(SD)	2100(1094)	1297(950)	2355(1380)	0.003*
Serum Creatinine(mg/dl)(IQR)	1.81(1.5–2.5)	2.23(1.68–3.45)	0.78(0.56–0.86)	<0.001*
AKI stage(%)				
Stage1	41(54)	20(30)	0	0.02*
Stage2	20(25)	23(34)	0	0.3
Stage3	16(20)	24(36)	0	0.05*
Strong ion difference (mEq/l)(SD)				
SIDu	30(24)	47.3(21.5)	16.4(12)	<0.001*
SIDa	38.8(6.8)	38.2(4.9)	38.3(3.2)	0.99

Results reported as means(SD), medians(interquartile range) or n(%).p values are for comparison across the three groups.

* is for variables that differ statistically across groups of patients.

**Fig 2:**
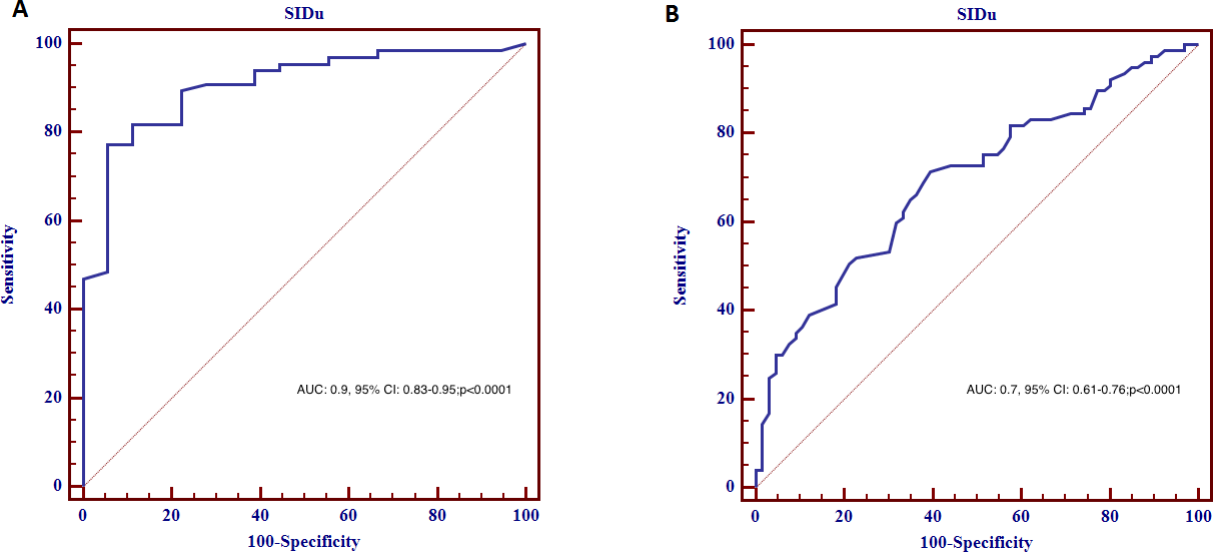
**A**. Receiver Operating Characteristic curve for day-0 urinary Strong Ion Difference(SIDu) to discriminate controls (no-AKI) and P-AKI patients. **B**. Receiver Operating Characteristic curve for day-0 urinary Strong Ion Difference(SIDu) to discriminate R-AKI and P-AKI patients.

The diagnostic performance of Day-0 SIDu in discriminating controls and P-AKI patients was excellent (AUC:0.9, 95% CI:0.83–0.95; p<0.0001) (Fig 2A). A cut-off of 30.8 mEq/l had the highest sensitivity and specificity for the examined purpose (77% and 94% respectively). The diagnostic performance of Day-0 SIDu in discriminating R-AKI and P-AKI patients was fair (AUC: 0.7, 95% CI: 0.61–0.76; p<0.0001)(Fig 2B). A cut-off of 40 mEq/l had the highest sensitivity and specificity for the examined purpose (72% and 60% respectively)

## Discussion

Urinary output and blood biomarkers such as SCr are complementary tools in AKI evaluation. However, their role has been questioned by many[13]. Recently, new emphasis has been placed on urine and acid-base status in monitoring the decelopment of AKI[18,24]. In our study, we evaluated SIDu values in patients with and without AKI. AKI patients were classified as having R- or P-AKI. Although questioned by some authors, this temporal distinction has been shown to have a prognostic validity[23,25], as it identifies two groups of patients with different renal function and prognosis. Our results suggest that SIDu may be of prognostic value in patients with AKI, as higher SIDu values were observed in patients with P-AKI. SIDu reflects the physiologic drive for body fluid electroneutrality[15]. Little is known about the role of SIDu as a marker of renal dysfunction, with most studies analyzing patients with renal dysfunction and concomitant metabolic acidosis[20,21]. Kellum proposed that an adequate response to non-renal metabolic acidosis should be a negative SIDu[19]. When a strong acid is added to plasma, plasma SID decreases and metabolic acidosis results. In this setting, renal compensation is marked by increases in NH_4_Cl excretion, which allows the elimination of Cl^-^ with a weak cation. Consequently, SIDu becomes negative, thus increasing the plasma SID with a net alkalizing effect. Moviat et al. examined the plasma and urine chemistry in 65 critically ill (mixed medical and surgical) patients with metabolic acidosis. They found that in patients with metabolic acidosis, impaired renal function was associated with greater urinary SIDs[22].

In the present study, we evaluated SIDu values in AKI and controls. SIDu values differed when compared to patients with normal renal function, with rising values from controls to P-AKI groups. A similar behavior in SIDu values has been previously shown by Maciel et al., suggesting that alterations in NaU and ClU values may be viewed as part of AKI development in critically ill patients[23]. In this setting, a defect of urine acidification seems to be characteristic of AKI. In addition, day-0 SIDu diagnostic performance in discriminating controls and P-AKI was excellent, while it performed less well in discriminating R-AKI and P-AKI groups.

This study has several limitations that deserve mention. First, this study is a retrospective analysis that did not allow control of several variables and patients’ inclusion was dictated by the availability of urine samples for SIDu estimation. As a result, patients' inclusion did not necessarily correspond to AKI onset, potentially grouping together patients by similar KDIGO stage who were at different time points in AKI evolution.

Second, the study population was extremely heterogeneous. Different AKI causes and pathophysiologic bases could have led to different urine biochemistry profiles. The small study population precluded subgroup analysis.

Third, SIDu is one of the three independent variables that determine acid-base balance. Its determination should be considered in light of other variables, such as pH, SIDa, SIDe, type and SID of infused fluids. Available data did not allow us to analyze these variables, potentially limiting the applicability of our finidngs.

Last, diuretic therapy is known to influence urine composition and acid-base status. As such, loop diuretics increase Na^+^U and Cl^-^U concentrations and decrease SIDu in patients with normal renal function, with a net alkalinizing effect[17,26]. Diuretic therapy could be a confounding factor when interpreting urine electrolyte composition. However, as showed in our population, the highest diuretic doses were administered to patients with the most severe renal dysfunction and it is possible that diuretics might have had less impact on SIDu derangements. This question remains to be specifically evaluated.

## Conclusion

SIDu may be a promising, simple and inexpensive tool in the’ evaluation of patients with AKI. Further research is needed to assess the utility of SIDu in the early detection of patients with renal dysfunction prior to increases in serum creatinine or decreases in urine output.
